# Long-Term Outcome of Autotransplantation of a Complete Root Formed a Mandibular Third Molar

**DOI:** 10.1155/2021/5512804

**Published:** 2021-11-27

**Authors:** Hiroyuki Kimura, Yusuke Hamada, Taro Eida, Tsuyoshi Kumano, Kazutoshi Okamura, Makoto Yokota

**Affiliations:** ^1^Private Practice, Kumamoto, Kyushu, Japan; ^2^Department of Periodontology, Indiana University School of Dentistry, Indianapolis, IN, USA; ^3^Faculty of Dental Science, Kyushu University, Japan; ^4^YDA Yokota Juku, Japan

## Abstract

Autogenous tooth transplantation is a procedure to reposition an autogenous tooth to another extraction area or surgically created recipient site. The autotransplantation procedures have been documented well in the literature, and the survival rate of the transplanted teeth was reported to be more than 90% after ten years. Therefore, autotransplantation might have been overlooked as a treatment option. The purpose of this case report is to evaluate the long-term (29-year) success and periodontal stability of the tooth autotransplantation from the mandibular third molar to the second molar. A 24-year old female presented to a clinic with a large caries lesion with periapical radiolucnecy on to tooth #18. The tooth was extracted with the site and treated with autogenous tooth transplantation from #17 with a complete root form. Endodontic treatment was completed 3 months post autotransplantation; the final prosthesis was placed 6 months postoperatively. The patient has shown excellent oral hygiene care and high compliance with the regular maintenance recall program. The transplanted tooth has been still functioning without any symptoms. Radiographic and clinical examinations revealed stable periodontal and endodontic conditions over the 29 years after the procedure. This case report showed the long-term success of autotransplantation of the mandibular third molar with a closed root apex to the second molar site. Autotransplantation can be an option when an adequate donor site is available to reconstruct the occlusion after the tooth extraction.

## 1. Introduction

One of the dental treatment goals is reconstructing the partial and complete edentulism with satisfactory functional and esthetical outcomes. Although dental implants and fixed prostheses have been utilized to replace the missing teeth, tooth autotransplantation can be a viable option to reestablish stable occlusion when an appropriate donor site is available [1, 2]. Autogenous tooth transplantation is a procedure to reposition an autogenous tooth to another extraction area or surgically created recipient site [3, 4]. The transplantation of the teeth has multiple benefits compared to other treatment modalities such as dental implants or fixed prosthesis. The advantages include that the procedure is indicated to children and young adults who have not completed the maxillofacial growth [5]. Furthermore, the transplanted tooth can stimulate alveolar bone growth with the eruption process due to the presence of periodontal ligament. Besides, the transplanted teeth can be moved to the ideal position with orthodontic treatment if necessary [5, 6]. However, multiple factors affecting results need to be taken into account with the procedure, such as surgeons' skill and knowledge, patient selections, local inflammatory status, endodontic treatments, and availability of periodontal ligament in both donor and recipient sites [7–10]. The autotransplantation procedures have been documented well, and systematic reviews and meta-analysis showed that the survival rate of the transplanted teeth was more than 90% after ten years [8, 11]. Moreover, a systematic review by Machado et al. included studies that had more than six years of follow-up period to analyze the long-term prognosis [7]. The meta-analysis revealed an 81% survival rate, and this rate showed an excellent long-term therapeutic prognosis of autotransplantation.

However, the evidence of the very long-term outcome of autotransplantation is still limited. Therefore, this case report is aimed at demonstrating the 29-year follow-up of the successful autotransplantation of the mandibular third molar to the second molar.

## 2. Clinical Presentation

**Figure 1:**
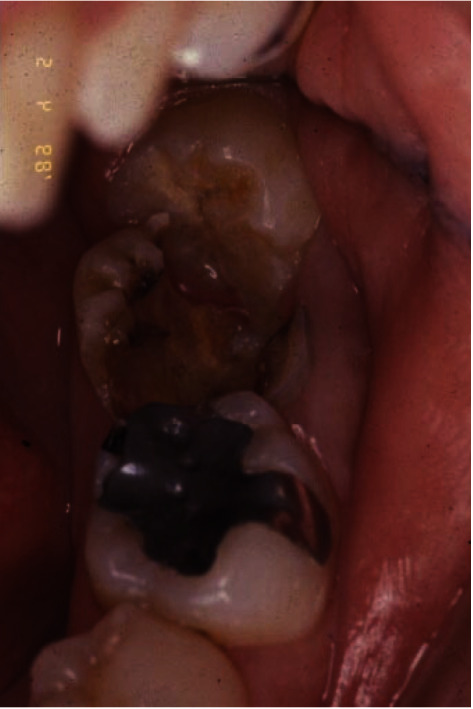
The initial clinical presentation of #18 with gross caries extending to the subgingival margin.

**Figure 2:**
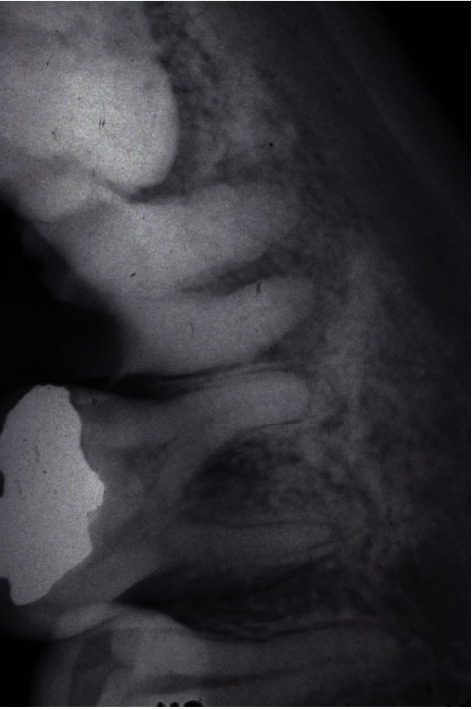
Periapical radiograph at the initial appointment. Large periapical radiolucency was present on the second molar. The gross caries reached to the almost alveolar bone level on the radiograph. This was deemed as a nonrestorable tooth.

**Figure 3:**
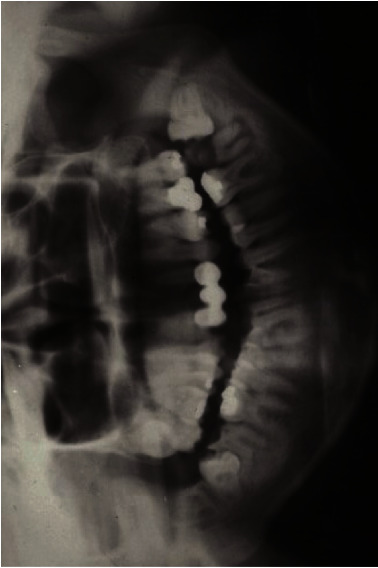
Panoramic radiograph showed that horizontal impaction of #17 was noted. The apical part of #17 was fully formed.

A 24-year-old Asian female presented to private practice on March 29, 1989. She was with the chief complaint of caries treatment in the lower left second molar. She was classified as ASA I, and no history of smoking was noted. Tooth #18 was considered as a nonrestorable tooth based on the clinical and periapical radiograph (Figures 1 and 2). Panoramic radiograph showed large periapical radiolucency on #18 and relatively a conical shape of the #17 root form (Figure 3). Since she was not interested in having a dental implant at the time, this patient chose autologous tooth transplantation from #17 to #18.

**Figure 4:**
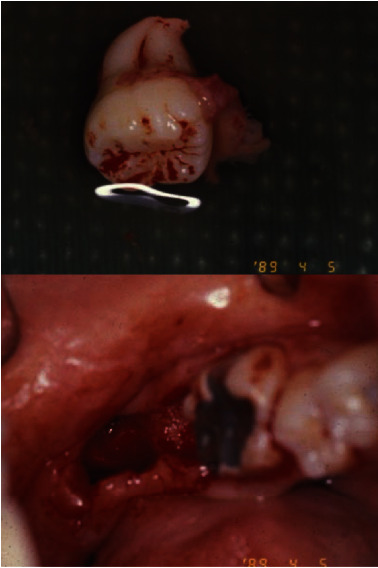
Immediate after of minimal traumatic extractions of #17 and 18. #17 was kept in the saline until this was used.

**Figure 5:**
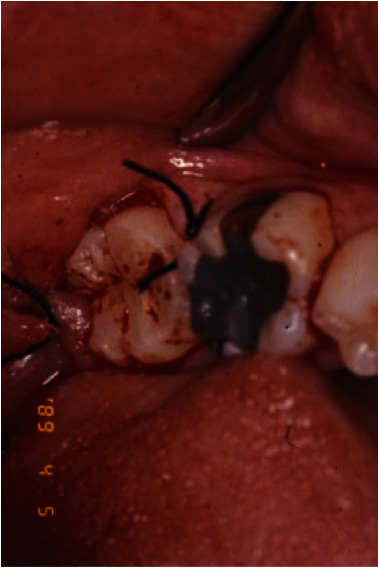
Tooth #17 was transplanted into the #18 site. Interrupted sutures were rendered to proximate the gingival tissues.

The patient was fully informed of all possible adverse events, and she consented to the procedure before the procedure. The procedure was completed under local anesthesia in April 1989. Initially, #18 was extracted in a minimal traumatic manner and meticulous debridement was completed in the socket. Following the extraction of #17 with the caution of saving periodontal regiment, the removed #17 was temporarily stored in a glass petri dish with 0.9% of saline solution (Figure 4). The recipient site was prepared with a low-speed handpiece (20,000 rpm) with carbide round bur under copious irrigation. This process was repeated until the root shape of #17 fit well in the recipient site. Since the transplanted tooth did not show any mobility after the insertion, the temporary fixation was completed with the 4-0 silk sutures in an interrupted manner. The total duration from extraction to stabilization of #17 in the recipient site was less than 10 minutes. An occlusal adjustment was completed to remove any contacts to the opposing arch on the transplanted tooth (Figure 5). This patient was prescribed bacampicillin 250 mg and diclofenac 25 mg three times a day for three days and was instructed to refrain from using the left side for mastication. Suture removal was completed one week postoperatively. Approximately 3 months after the surgical procedure, the swelling was noted on the buccal surface of the tooth. The transplanted tooth was diagnosed with pulp necrosis. On the same day, endodontic treatment was rendered. Since periodontal tissue was fully stabilized within 6 months after the surgical procedure, the final prosthesis was delivered on the transplanted tooth in October 1989.

**Figure 6:**
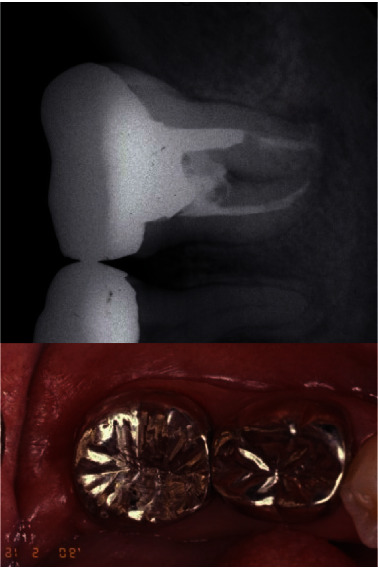
One year follow-up from the surgical procedure. Slight apical radiolucency was noted in the transplanted tooth. Soft tissue healing was uneventful. Slight inadequate root canal filing material was noted on the distal root of the transplanted tooth.

**Figure 7:**
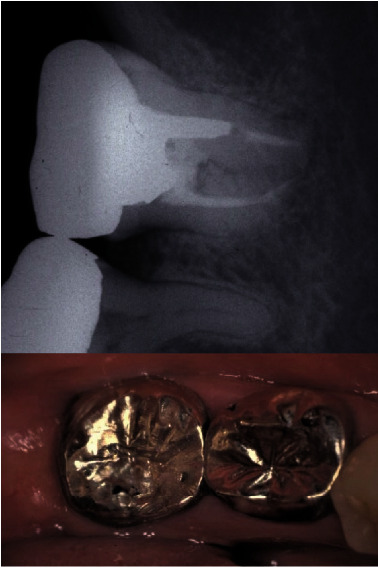
Ten-year follow-up after the transplantation. The size of the apical radiolucency reduced from one year after the procedure.

**Figure 8:**
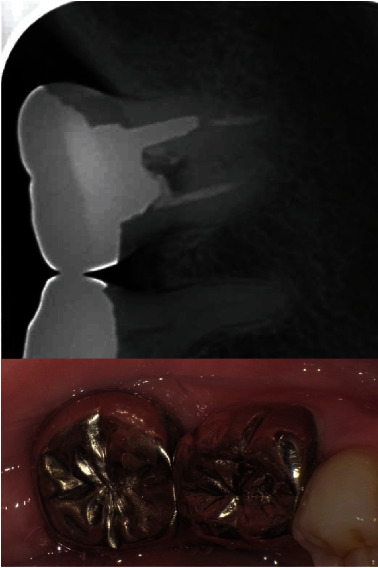
Twenty-eight years after the transplantation. Probing depth was less than 3 mm and gingival health was confirmed. There is no alveolar bone loss that was noted around the transplanted tooth. A radiolucency was noted in the pulp chamber which reached to the furcation area. However, no clinical pathological changes were noted.

**Figure 9:**
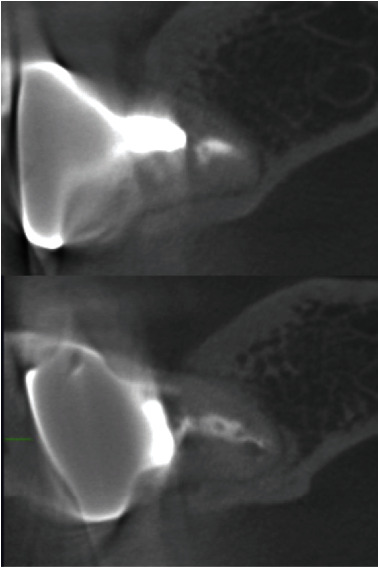
CBCT revealed the presence of buccal and lingual bone on both mesial (a) and distal (b) roots of the transplanted tooth.

One year following the autotransplantation, slight periapical radiolucency was still noted. However, since the tooth was not symptomatic and probing depth was within 3–4 mm without any alveolar bone loss, the maintenance and oral hygiene program began one year postoperatively. Slightly inadequate root canal filling material was noted on the distal root of the transplanted tooth (Figure 6). A periapical radiograph and clinical examinations showed reduced periapical radiolucency and periodontal stability ten years after the procedure (Figure 7). At the 28-year follow-up in 2017, probing depth around the tooth showed within 3 mm without bleeding on probing and the interdental bone level was within 2 mm from cement enamel junction (Figure 8). Cone beam computed tomography (CBCT) showed the absence of root resorption and the presence of buccal and lingual bone on the transplanted tooth (Figure 9).

**Figure 10:**
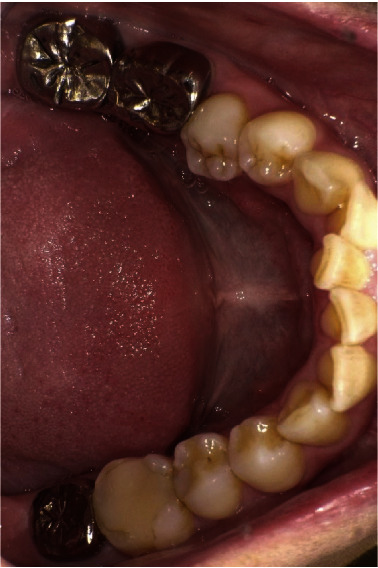
Twenty-nine years after the procedure, this patient maintains good oral hygiene and gingival health.

**Figure 11:**
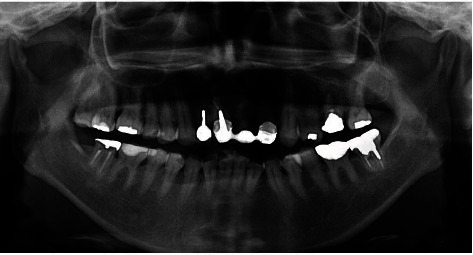
Panoramic radiograph demonstrated the no sign of alveolar bone loss on the transplanted tooth. This patient has maintained all the teeth for almost 30 years from initial appointment.

After 29 years of the procedure, in February 2018, the transplanted tooth was still functioning without any discomfort or symptoms (Figures 10 and 11). This patient has complied every 4–6 months of the maintenance program, and occlusal check and adjustment have been rendered if necessary, at each visit. The patient was delighted with the outcome of the treatments over these years.

## 3. Discussion

This case report demonstrated the long-term success and periodontal stability of the transplanted tooth. Even with this patient's age and suitability of a dental implant on #18 after the extraction, the autotransplantation was attempted because this procedure included a single surgical intervention; the tooth presented a conical root shape, even if the root form was almost completed. The total cost of autotransplantation can be lower compared to that of implants because the procedure is performed in one stage, and a prosthesis may not be needed in some cases [12]. The predictability of the procedure needs to be considered for the choice of treatments. Fugazzotto reported that the cumulative success rate of implants in the mandibular second molar was 85% and the rate was slightly lower than other molars [13]. On the other hand, the total success rates of autotransplantation were 94% with the open apex teeth and 84% with closed apex groups [14]. The other retrospective study showed 92% of the survival rate of the immature root form-autotransplanted teeth for midterm length [15]. In this study, the authors utilized the enamel matrix derivatives (EMD) to apply the root surface to enhance the healing process if necessary. The additional biologic modifiers might play a crucial role in regenerative therapy. Based on these previously reported high predictabilities, the toot autotransplantation should be considered as one of the treatment options when an adequate donor site is available. However, the autotransplantation procedure is not complication free. Unsuccessful autotransplantation is more likely associated with excessive surgical trauma and contaminated donor tooth and when the periodontal probing depth is more than 4 mm and patients are older than 40 years [16]. A retrospective study reported that the main reasons for autotransplantation failure were periodontal attachment loss (54.9%), root resorption (26.5%), dental caries (4.0%), and root fracture (2.9%) [17]. In this case, the patient was 24 years old at the time of the procedure and did not present evidence of periodontitis. Patient selection factors, including stable systemic conditions, excellent oral hygiene, and high compliance with the regular dental visits with occlusal adjustments, play essential roles in achieving the ideal outcomes.
